# Effect of encapsulation technology and *in vitro* digestion on the hypocholesterolemic activity of *Limosilactobacillus fermentum* K73

**DOI:** 10.3389/fmicb.2025.1622211

**Published:** 2025-08-29

**Authors:** Mary Cardoso-Cardenas, Katherine Bauer-Estrada, Luis Eduardo Diaz-Barrera, Maria Ximena Quintanilla-Carvajal

**Affiliations:** ^1^Agroindustrial Processes Research Group, Universidad de La Sabana, Engineering Department, Chía, Colombia; ^2^Unisabana Center for Translational Science, Universidad de La Sabana, School of Medicine, Chía, Colombia

**Keywords:** probiotic, spray-drying, BSH, cell adhesion, Lactobacillaceae

## Abstract

**Introduction:**

Members of the Lactobacillaceae family have been extensively investigated for their health-promoting properties, including the restoration of cellular functions, inhibition of pathogen colonization, and cholesterol-lowering effects.

**Methodology:**

This study evaluated the hypocholesterolemic activity of *Limosilactobacillus fermentum* K73 at various stages of encapsulation using three distinct capsule types, including simulated gastrointestinal (GI) transit and analysis of adhesion to HT-29 and RKO intestinal epithelial cells.

**Results:**

The strain demonstrated a pronounced ability to deconjugate taurine-conjugated bile salts, particularly taurocholic acid sodium (TCA) and sodium taurodeoxycholate acid (TDCA), as evidenced by viable cell counts. In bioreactor samples, counts reached 8.31 and 7.82 log CFU/mL for TCA and TDCA, respectively. After capsule dissolution, viability decreased across all formulations: C1 showed 6.90 and 5.04, C2 reached 4.99 and 4.68, and C3 recorded 6.10 and 4.12 log CFU/mL. Following *in vitro* digestion, C1 exhibited notable recovery, with 7.30 log CFU/mL (TCA) and 4.26 log CFU/mL (TDCA). Cholesterol absorption was highest in digested C1 (65.49%), surpassing the 24-h broth culture (63.9%). Adhesion to epithelial cells varied by formulation; for HT-29 cells, C1, C2, and C3 showed 21, 5, and 33 adhered bacteria, respectively, while for RKO cells, adhesion was 44 (C1), 22 (C2), and 201 (C3).

**Discussion:**

These findings demonstrate that *L. fermentum* K73 maintained its viability, hypocholesterolemic activity, and epithelial adhesion capacity throughout encapsulation and simulated GI digestion, supporting its potential application as a functional probiotic strain in nutraceutical and food products.

## 1 Introduction

Probiotic microorganisms have been extensively studied in recent decades due to their diverse health-promoting effects, their interaction with the intestinal microbiota, and the beneficial compounds they produce during fermentation (Latif et al., 2023). These microorganisms contribute to host health by modulating immune responses, enhancing gut barrier integrity, and exerting antimicrobial effects, making them highly relevant in clinical and nutritional contexts.

Among the various functional properties of probiotics, their hypocholesterolemic activity is particularly notable. This effect can be mediated through several mechanisms: (i) bile salt deconjugation by bile salt hydrolase (BSH), which reduces bile acid reabsorption and promotes cholesterol catabolism; (ii) cholesterol assimilation and conversion to coprostanol, a less absorbable derivative that lowers serum cholesterol levels (Lye et al., 2010); and (iii) adsorption of cholesterol to the bacterial cell membrane, limiting its absorption in the intestine (Kimoto et al., 2002).

*Limosilactobacillus fermentum* K73 has demonstrated a significant hypocholesterolemic potential through BSH-mediated bile salt deconjugation (Cueto and Aragón, 2012). However, to exert these effects *in vivo*, the strain must survive gastrointestinal (GI) transit and reach the intestine in viable and functional form, in sufficient numbers to interact with dietary cholesterol (Puttarat et al., 2023).

The harsh conditions of the gastrointestinal tract, such as acidic pH, bile salts, and digestive enzymes, can compromise the viability and functionality of probiotics. In this context, encapsulation technologies play a crucial role by protecting probiotics during GI transit and storage and enhancing their delivery and stability in food or pharmaceutical matrices (Rajam and Subramanian, 2022; Vivek et al., 2023).

Two widely used encapsulation techniques are spray drying (SD) and ionic gelation (IG). SD involves atomizing a probiotic-containing suspension into a hot air stream to obtain a dry powder. It offers several advantages, including scalability, cost-effectiveness, and high process reproducibility (Mohammed et al., 2020; Gullifa et al., 2023). IG, in contrast, is a gentle encapsulation method that avoids high temperatures and organic solvents. It forms gel matrices via ionic crosslinking, suitable for hydrophilic and hydrophobic compounds, and allows for controlled release (Da Fonseca et al., 2020; Bennacef et al., 2021).

Moreover, incorporating oils rich in unsaturated fatty acids during encapsulation has shown potential to mitigate oxidative stress, thereby enhancing probiotic survival. These oils and antioxidant phenolic compounds protect cellular components—including lipids, proteins, and nucleic acids—against damage induced by reactive oxygen species (ROS) generated during processing (Jiang et al., 2022).

To evaluate the functionality of encapsulated probiotics after exposure to simulated GI conditions, standardized *in vitro* digestion protocols are essential. The INFOGEST protocol, developed by an international consortium, offers a validated static model that mimics oral, gastric, and intestinal phases, facilitating reliable and comparable assessments of nutrient and bioactive compound bioaccessibility.

Beyond survival, the adhesion of probiotics to intestinal epithelial cells is essential for their efficacy. Adhesion enables colonization, modulation of immune responses, competitive exclusion of pathogens, and reinforcement of the gut barrier (Monteagudo-Mera et al., 2019). Additionally, adhesive probiotics contribute to the production of beneficial metabolites, such as short-chain fatty acids, further supporting gut health (Markowiak-Kopeć and Śliżewska, 2020).

Given the multifactorial nature of probiotic functionality, preserving their biological activity throughout production, storage, and consumption is critical. While encapsulation technologies have shown promise, significant knowledge gaps remain regarding how specific production and formulation processes affect probiotic viability and activity (Dos Santos Nascimento et al., 2024; Moraffah et al., 2025).

The significance of microorganisms with probiotic properties is well established, as evidenced by their numerous health benefits to the host (Ouarabi et al., 2025). These benefits include the mitigation of allergic responses, reduction of serum cholesterol levels, notable anti-inflammatory activity, antitumor and antibacterial effects, and the prevention of gastrointestinal inflammatory disorders (Rousseaux et al., 2023; Sudheer et al., 2025).

Given the relevance of these biological activities, preserving the functionality of probiotics is essential. In this context, biotechnological and process engineering tools play a pivotal role. These disciplines support the development of advanced methodologies and technologies aimed at designing functional food ingredients and nutritional supplements. A critical aspect of this process is ensuring that probiotics retain their viability and biological activity until the point of consumption (Dos Santos Nascimento et al., 2024; Moraffah et al., 2025).

Despite considerable research on probiotic functionality and the technologies supporting their application, a significant knowledge gap persists—specifically, regarding the impact of production and formulation processes on the probiotic itself. This study seeks to address this gap by evaluating strategies to preserve the biological activity of the probiotic microorganism throughout processing, with the ultimate goal of ensuring the delivery of its health-promoting properties to the consumer.

Therefore, this study aims to evaluate the hypocholesterolemic potential of *L. fermentum* K73, focusing on its enzymatic activity and cholesterol uptake under different encapsulation conditions. The study further investigates its survival through simulated digestion using the INFOGEST model, and its adhesion to HT-29 and RKO intestinal cell lines post-digestion. The findings will contribute to a deeper understanding of how encapsulation and gastrointestinal simulation influence probiotic efficacy, ultimately informing the development of more robust and effective probiotic-based interventions. These results are expected to support the rational design of encapsulated probiotics for application in functional foods targeting cholesterol regulation and gut health.

## 2 Materials and methods

### 2.1 Production of *Limosilactobacillus fermentum* K73

Production of *L. fermentum* K73 biomass was performed following the protocol described by Aragón-Rojas et al. (2018). Briefly, the biomass of bioreactors was produced by inoculating MRS broth at 10% (v/v) with *L. fermentum* K73 and incubating at 37 °C for 12 h. After that, a bioreactor of 1 L (Bioflo 110, New Brunswick Scientific Co, Inc., United States) with culture medium prepared with 8% whey and 0.22% yeast extract, adjusted to pH 5.5 (sterilized at 121 °C for 15 min) was inoculated with the previously prepared inoculum and fermented during 10 h at 37 °C and 100 rpm.

### 2.2 Encapsulation of *Limosilactobacillus fermentum* K73

#### 2.2.1 Microencapsulate 1 (C1)

Microencapsulate 1 (C1) was obtained following the methodology proposed by Bauer Estrada et al. (2025). After fermentation, *L. fermentum* K73 was concentrated and emulsified with high oleic palm oil (HOPO) in an Ultraturrax at 15 xg. Then, the emulsion obtained was dispersed into a solution of whey in water (31% w/w) and then dried in a spray drier at an inlet temperature between 170 and 200 °C, with a delta T of 110 °C.

#### 2.2.2 Microencapsulate 2 (C2)

Microencapsulate 2 (C2) production was carried out using a similar composition of C1 but without the use of the HOPO, whose composition is based on sweet whey, maltodextrin, and the fermentation product of *L. fermentum* K73 (Aragón-Rojas et al., 2020). The sealing of the capsule was performed in a pilot scale spray drier with operating conditions of 175 °C inlet temperature, 80 °C outlet temperature, and 1 bar pressure.

#### 2.2.3 Microencapsulate 3 (C3)

Microencapsulate 3 C3 was produced accordingly by Rojas-Muñoz et al. (2023) in a Buchi encapsulation system. Sodium alginate, whey, and the fermentation product of *L. fermentum* K73 were the components of this microcapsule. The operating conditions were found in the range of 68–72 Hz, 200–300 V, 10–30 mL flow rate, and 300 μm nozzle (Rojas-Muñoz et al., 2023).

### 2.3 Viability of probiotic

The viability of the probiotic was measured by plate counting. 1 g of microcapsules were decapsulated, plated in duplicate on MRS agar, and incubated at 37 °C for 24 h. Colonies were then counted and expressed as log CFU/mL (Aragón-Rojas et al., 2019).

The following formula was used to calculate the CFU/mL:


$$\mathrm{CFU}/\mathrm{mL}=\frac{\text{Average count}*\text{Dilution factor}\;}{\text{Sample volume}\;(\mathrm{mL})}$$

In order to express the results of microbial viability in a more concise and manageable way, the values obtained in colony-forming units per milliliter (CFU/mL) were transformed to their logarithm in base 10 (log CFU/mL). The result of CFU/mL was then calculated as the logarithm in base 10 to obtain the result in log CFU/mL.

### 2.4 Measurement of powder properties

#### 2.4.1 Moisture content

Moisture content was measured using 2 g of powder in a Mettler LJ16 thermobalance (Mettler Toledo, Switzerland).

#### 2.4.2 Water activity

Water activity was measured using AquaLab equipment (Decagon Devices, Inc., United States) using the dew point method at 25 °C for 30 min.

#### 2.4.3 Bulk density

The bulk density was determined following the method outlined by Su et al. (2023). To this end, 1 g of powder was weighed into an empty 10 mL graduated cylinder, and the powder particles were distributed evenly. The graduated cylinder was then placed on a solid, flat surface, and the volume of the powder was measured. The bulk density was then calculated as the ratio of the mass of the powder to the volume occupied in the cylinder (g/mL).

#### 2.4.4 Solubility

Solubility was determined following the method described by Ricaurte et al. (2017)1 g of powder was weighed and dissolved in 50 mL of distilled water, with agitation maintained at a constant rate of 850 rpm. The duration required for complete dissolution was measured in seconds (s).

### 2.5 *In vitro* digestion assays

The viability of the encapsulated microorganisms through *in vitro* digestion was performed following the standardized INFOGEST protocol proposed by Minekus et al. (2014). The three phases of digestion were performed for all encapsulates: oral, gastric, and intestinal. In the oral phase, 1 g of each powder was mixed with simulated salivary fluid (SSF) in a 1:1 ratio (w/v) without adding *α*-amylase, since the microcapsules did not contain starch, and kept under stirring for 2 min at 37 °C. At the end of the oral phase, it was mixed with simulated gastric fluid (SGF) and adjusted to pH 3 with HCl; in this phase, porcine pepsin was added to reach 2000 U/mg (Sigma, P6887-5G), and the gastric phase was stirred for 2 h at 37 °C. Finally, the intestinal phase was obtained by mixing the solution obtained in the gastric phase with simulated intestinal fluid (SIF) containing bile extract (Sigma, B8631-100G), trypsin (Sigma, P7545-25G), and lipase (L3126-100G), to reach final concentrations of 10 mM, 100 U/mL, and 2000 U/mL, respectively. The pH was adjusted to 7 with 1 M NaOH and stirred at 37 °C for 2 h.

After each phase, 100 μL aliquots were collected to measure the viability of the probiotic. The methodology for this measurement is described in section 2.3.

### 2.6 Biochemical assessment of hypocholesterolemic activity

The evaluation of the hypocholesterolemic activity of probiotic *L. fermentum* K73 was carried out at 4 different points of the process: (i) after 24 h of growth in MRS broth, (ii) after 10 h of fermentation in the bioreactor with the medium composed of whey; (iii) after the three encapsulation processes (C1, C2 and C3); (iv) the C1 microcapsule was subjected to *in vitro* digestion.

#### 2.6.1 Semi-qualitative assessment of the presence of BSH enzyme

A semi-qualiitative analysis was conducted using the methodology outlined by Huang et al. (2019), with minor adaptations. The enzymatic activity was evaluated through the bioavailability of the microorganism in an MRS medium supplemented with bile salts. The procedure was conducted in a series of tenfold dilutions. An aliquot was taken from each point to be analyzed, and plated by duplicate on MRS agar supplemented with 0.5% (w/v) sodium taurodeoxycholate acid >95% (TDCA, Sigma T0875-5G), taurocholic acid sodium >95% (TCA, Sigma T4009-5G), sodium glycocholate >95% (GCA, Sigma G7132-5G), and 0.37 g/L CaCl₂. Then agar plates were incubated at 37 °C for 72 h in anaerobic conditions, with a separate medium utilized for each bile salt. The colony formation units per milliliter (CFU/mL) were calculated. Additionally, negative control was included in the analysis, whereby the same procedure was conducted using an MRS medium without supplementation. This negative control allowed for a comparison with the regular growth conditions of the microorganism.

#### 2.6.2 Cholesterol absorption

The cholesterol absorption occurred at the following points: (i) after 24 h of growth in MRS broth, (ii) after 10 h of fermentation in the bioreactor with the medium composed of whey; (iii) after the three encapsulation processes (C1, C2 and C3); (iv) The C1 microcapsule was subjected to *in vitro* digestion.

Cholesterol absorption was carried out according to the methodology proposed by Aragón Rojas (2012), as follows: *L. fermentum* K73 was inoculated at 1% in MRS broth supplemented with 0.2% (w/v) of thioglycolate, 0.2% (w/v) of taurocholic acid sodium >95% and sodium glycocholate >95%, with the addition of cholesterol solution >99% (Sigma C3045-25G) and incubated under anaerobic conditions; after incubation, it was centrifuged at 7600 g for 15 min at 4 °C. The supernatant was used to measure the concentration of remaining cholesterol in the broth. An aliquot of the supernatant was mixed with KOH (33%, w/v) and ethanol (96%) by vortexing, heated to 60 °C for 15 min, and cooled to 25 °C. Hexane was added to extract the cholesterol and then concentrated by Rota-evaporation in a Hei-VAP Precision roto evaporator (Heidolph, Germany) at 100 mbar, 100 rpm rotation at 37 °C. Then, o-phthalaldehyde reagent (50 mg/dL) was added to dissolve the remaining residue, and 98% sulfuric acid was added to the mixture; after 90 min, the absorbance was observed in a spectrophotometer at 550 nm.

A calibration curve was established with cholesterol solutions with concentrations ranging from 10 to 70 μg/mL in duplicate, using the same extraction method described above and reading the absorbance at each point, to determine the concentration of cholesterol in the samples. The following equation was used to determine the percentage of cholesterol absorption:


$$\begin{array}{l} \text{Percentage of cholesterol absorbed}=\\ \frac{\text{Cholesterol extracted}\;\mathrm{\mu g}/\mathrm{mL}}{\text{Cholesterol total}\;\mathrm{\mu g}/\mathrm{mL}}x100 \end{array}$$

### 2.7 Molecular identification of the presence of the BSH enzyme

The following points were used to determine the molecular identification of the presence of the BSH enzyme: (i) after 24 h of growth in MRS broth, (ii) after 10 h of fermentation in the bioreactor with the medium composed of whey; (iii) Subsequent to the encapsulation process for C1, (iv) the C1 microcapsule was subjected to *in vitro* digestion.

RNA extraction of *L. fermentum* K73 in all the points mentioned above and to two more standard strains as controls (*L. fermentum* ATCC 9338) was carried out using TRIzol reagent, followed by cDNA transcription using the One Script Plus cDNA Synthesis Kit (commercial kit from abm), following their protocol, utilizing Random primers. Once the cDNA was obtained, primers specific for *Limosilactobacillus fermentum* BHS enzyme were employed using the conventional PCR technique, with the following conditions: One Tag 2x (BioLabs), forward primer 0.2 mM, reverse primer 0.2 mM, cDNA 5 ng/μL. The thermocycler conditions were set to an initial denaturation at 95 °C for 3 min, denaturation at 95 °C for 30 s, annealing at 60 °C for 45 s, and extension at 72 °C for 30 s. This was followed by a final extension at 72 °C for 3 min over 35 cycles, resulting in the production of a 628 bp fragment. Subsequently, the quality of amplified products was analyzed in 1.5% agarose gel, with the cDNA bands visualized using the Quantity One software (Bio-Rad) version 4.6.8.

### 2.8 Cell adhesion assays

The evaluation of probiotic adhesion to cells was performed as described by Archer et al. (2018). The cell lines in question were obtained from the American Type Culture Collection (ATCC) [HTB-38 HT-29, and (CRL-2577)] RKO cells derived from human colorectal adenocarcinoma. For this, the two tumor lines were maintained in Dulbecco’s modified Eagle’s minimal essential medium supplemented with 10% (v/v) of inactivated fetal bovine serum, penicillin (100 μg/mL) and streptomycin (100 μg/mL). Cells were incubated with 5% CO_2_ at 37 °C during all the assays. For the adhesion assay, 200 μL of the suspension of each cell line was grown in a 96-well plate to a density of 4 × 10^4^ cells/well and maintained under the same incubation conditions until a monolayer was formed. Then the cells were incubated for 2 h with eight different treatments by quadruplicate. (i) *L. fermentum* K73 growth in MRS broth for 24 h; (ii) Bioreactor after 10 h of fermentation *L. fermentum* K73 in a food grade medium; (iii) C1 dissolved; (iv) C2 dissolved; (v) C3 dissolved; (vi) C1 after intestinal phase; (vii) C2 after intestinal phase; (viii) C3 is digested after the intestinal phase.

After the incubation period, the cells were washed three times with PBS, fixed with methanol, and stained with Gram stain for microscopic examination. The count of adherent bacterial cells was performed as described by Chauviere et al. (1992), taking 20 microscopic fields at random from each treatment and counting each bacillus present in the analyzed area. This approach expressed probiotic adherence as the number of bacteria adhering to 100 HT-29 and RKO cells.

### 2.9 Statistical analysis

The R statistical software (version 4.4.3) was utilized to analyze and visualize the data. A one-way analysis of variance (ANOVA) was conducted, with a 95% confidence interval. Subsequently, Tukey’s post-hoc test for multiple comparisons was applied, considering a significance level of *p* < 0.05.

## 3 Results

### 3.1 Production of encapsulates of *Limosilactobacillus fermentum* K73

The measured properties showed that microencapsulates were reproducible. Briefly, the capsules presented lower values of moisture content (less than 5%), which improves the stability of the product during shelf life (Table 1). In addition, lower water activity values favor the quality of the encapsulates and the viability of the probiotic *L. fermentum* K73 contained therein. This can be compared with that reported by Aragón-Rojas et al. (2020), (C2) used in this work had moisture of <4% and water activity (aw) of <0.2, on the other hand, according to the authors Vargas et al. (2024) and Agudelo-Chaparro et al. (2022) with another type of encapsulation with maltodextrin, the water activity fluctuated between 0.23 and 0.44 and 0.16 to 0.54, respectively.

**Table 1:** Measurement of properties; stability of the C1 emulsion and the three types of encapsulations.

Sample	Property	Value
C1 emulsion	Potential z	−28.7 mV
	Drop size	471.6 nm
C1	Moisture	3.51%
	Water activity	0.193 aw
	Bulk density	0.5 g/mL
	Solubility	321.5 s
C2	Moisture	3.75%
	Water activity	0.29 aw
	Bulk density	0.5 g/mL
	Solubility	242 s
C3	Moisture	97.7%
	Size	1.8 mm
	Homogeneity	86%

It has been demonstrated that a product’s water activity (Aw) and moisture content are critical factors that significantly influence microbial stability and viability. Specifically, aw values below 0.61 have been shown to inhibit the growth of pathogenic microorganisms due to limited water availability (Tapía et al., 2020).

However, in the context of probiotic microcapsules, particularly those of *Lactobacillus* species obtained by spray drying, the relationship between water activity (Aw) and survival is more nuanced. Optimal probiotic survival during storage at room temperature has been reported over an aw range of 0.11 to 0.76 (Liu et al., 2016). This observation is consistent with the findings of studies indicating a significant loss of viability in products containing *Lactobacillus rhamnosus* with an aw of 0.938 after 28 days of storage at room temperature (Zavaleta et al., 2022).

On the other hand, C3 has a homogeneous size among the beads, obtaining an average of 1.8 ± 0.3 mm, a size like that reported by Rojas-Muñoz, Santagapita and Quintanilla-Carvajal, (2023), where they obtained beads with a size of 1.6 ± 0.2 mm. Also, it was observed that 86% of the beads had a circular shape without presenting irregular edges.

### 3.2 Viability of *Limosilactobacillus fermentum* K73

The production of *L. fermentum* K73 biomass reached a concentration of 8.41 ± 0,29 log CFU/mL after 10 h of the fermentation process. After this, three encapsulation systems were produced accordingly to Bauer Estrada et al. (2025), Aragón-Rojas et al. (2020), and Rojas-Muñoz et al. (2023). The viability of *L. fermentum* K73 after the production of each one of the microencapsulates was 6.99 ± 0,55, 6.45 ± 0,21, and 8.25 ± 0,49 log CFU/mL, for C1, C2, and C3, respectively.

Compared to the bioreactor, the C1 showed a 1.42 log CFU/mL reduction in viable cell count, decreasing from 8.41 to 6.99 log CFU/mL. This loss, although present, is notably lower than in other formulations and may be attributed to the protective effect provided by the encapsulation design, particularly the inclusion of HOPO oil. The incorporation of oil likely contributed to a more robust barrier against external stressors during the encapsulation process and subsequent gastrointestinal simulation. This observation aligns with findings reported by Yin et al. (2024), who demonstrated that using short-chain oils in double emulsion systems significantly enhanced probiotic survival rates from 43.23 to 65.16% by providing an additional protective layer during spray drying. The presence of oil in the formulation stabilizes the emulsion. It reinforces the encapsulation matrix, suggesting that oil-containing capsules, such as C1, offer superior protection and are more effective in preserving probiotic viability under challenging conditions.

The C2 formulation exhibited a viability loss of 1.96 log CFU/mL, decreasing from 8.41 to 6.45 log CFU/mL compared to the bioreactor, which can be compared to what is described by Bhagwat et al. (2020), as they proved that encapsulation with maltodextrin and whey as wall materials leads to loss of the viability of probiotics when they raise the outlet temperature of the spray dryer higher than 60 °C, causing cellular damage such as denaturation of genetic material, ribosomal damage and dehydration of cytoplasmic membranes of probiotics (Bhagwat et al., 2020). Similarly, Liu and collaborators explain that the use of maltodextrin can influence survival, as they observed that encapsulations with this material have a lower protective capacity for probiotics, due to its high solubility, generating a faster exposure of the microorganism to environmental and process conditions (Liu et al., 2019).

On the other hand, the encapsulation using sodium alginate and sweet whey to produce the C3 formulation demonstrated improved probiotic survival, with only a minimal viability loss of 0.16 log CFU/mL, decreasing from 8.41 to 8.25 log CFU/mL compared to the bioreactor. This claim can be substantiated by the encapsulation efficiency data presented by Vega-Carranza et al. (2021), which demonstrated the viability of approximately 99.97% ± 0.22% after encapsulation of *B. licheniformis*. In addition, this good encapsulation efficiency can be compared with the ones obtained by Raise et al. (2020), which presents high survival rates of the microorganism after *in vitro* digestion, showing that the technique favors the microorganism’s viability and gives it the necessary protection during passage through the gastrointestinal tract. It was observed that there was no contamination in any process.

Among the three encapsulation types evaluated, statistically significant differences in the viability of *L. fermentum* K73 were observed only in the C2 formulation when compared to the bioreactor. Tukey’s *post hoc* test revealed a reduction of 1.96 log CFU/mL in C2 relative to the bioreactor, with a statistically significant adjusted *p*-value of 0.0172. In contrast, the differences observed for C1 (−1.42 log CFU/mL, *p* = 0.0611) and C3 (−0.16 log CFU/mL, *p* = 0.9908) were not statistically significant.

After the encapsulation process, an *in vitro* digestion was performed to observe the behavior of the encapsulates in the gastrointestinal tract. Here, it was possible to observe that the three encapsulates did not lose logarithmic cycles when in contact with the oral phase (Table 2).

**Table 2:** MRS plate count in duplicate of the *in vitro* digestion expressed in log CFU/mL of *Limosilactobacillus fermentum* K73.

Digestion phase	Product	log CFU/mL
Oral	C1	7.04 ± 0.06
	C2	6.59 ± 0.02
	C3	6.69 ± 0.13
Gastric	C1	7.00 ± 0.12
	C2	5.76 ± 0.02
	C3	5.54 ± 0.09
Intestinal	C1	4.79 ± 0.02
	C2	4.59 ± 0.02
	C3	4.34 ± 0.62

On the other hand, when passing to the gastric phase with low pH, the C2 and C3 capsules lost only one cycle; on the contrary, the C1 encapsulated with palm oil remained stable. This phenomenon has been demonstrated in the study of Eratte et al. (2017), which revealed that the survival of *L. casei* increased in SGF when co-encapsulated with omega-3 oil, exhibiting a mere 1.5 log CFU/ml decrease. Likewise, although the three encapsulations use whey and the protein is denatured, flocculation was generated, which reduces the area of interaction between the protein and pepsin, thus reducing the protein digestion of the three encapsulations, in addition to the hydrophobic interaction generated between the aromatic amino acids, as presented by Aragón-Rojas et al. (2020). As stated in the work of Zhu et al. (2021) observations were made indicating that soy lecithin also favors protein flocculation. This is corroborated by what was found in this project since the release of the probiotic is done in the intestinal phase and not in the gastric phase, and C1 performs better than the other types of encapsulates (Table 2).

As for the intestinal phase, the three encapsulations showed reductions in viability of 5.08, 2.91, and 5.24 log CFU/mL for capsules C1, C2, and C3, respectively. This finding indicates that the encapsulation processes enable the probiotic to reach the intestine at a specific concentration. However, factors such as the stress induced by the pH shift between the phases and the activity of enzymes present in the intestinal phase can influence the probiotic’s survival, as previously reported by (Masco et al., 2007; Ranadheera et al., 2012).

### 3.3 Biochemical assessment of hypocholesterolemic activity of *Limosilactobacillus fermentum* K73

#### 3.3.1 Semi-qualitative evaluation of the presence of the enzyme BSH

**Figure 1:**
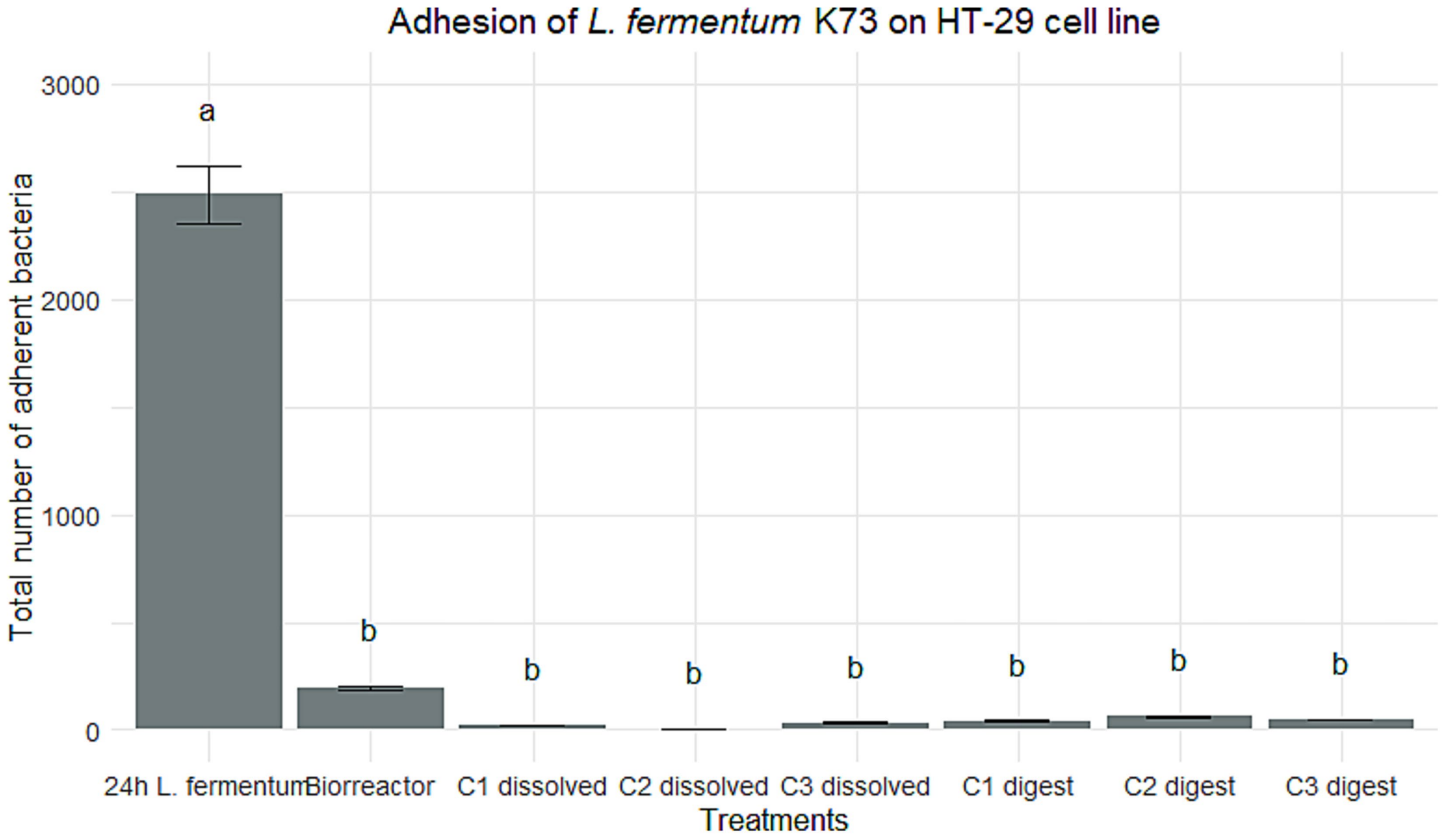
Comparison of *L. fermentum* growth in the presence of three types of bile salts (GCA, TCA, and TDCA), expressed in log CFU/mL, in different treatment conditions: broth culture for 24 h, bioreactor, dissolved C1, C2, and C3 capsules, and C1 after simulated digestion. Letters above the bars indicate statistically significant differences (*p* < 0.05) between treatments, determined by Tukey’s test.

The potential was evaluated by validating the activity of the enzyme bile salt hydrolase (BSH), based on the quantitative assay described above, during the four established processes. In most of the analyzed points, a decline in the growth of the microorganism was observed on MRS agar that had been supplemented with bile salts composed of sodium taurodeoxycholate acid (TDCA), sodium taurocholic acid (TCA), and sodium glucolate (GCA) (Figure 1). To analyze the behavior of the microorganism with the bile salts, a comparison was made with the negative control on normal MRS agar without supplementation.

Reduced growth was observed in the presence of GCA compared to TCA and TDCA, indicating a preferential affinity of *L. fermentum* K73 for tauroconjugated bile salts. This is consistent with previous findings by Dong and Lee (2018), who reported that structural features of *Lactobacillus* spp., particularly the presence of non-polar amino acids at terminal positions, enhance interaction with tauroconjugated substrates such as TCA and TDCA. This suggests that the strain possesses deconjugation capability and may also metabolize these substrates.

In the 24-h broth culture, although no statistically significant differences were observed among the bile salts, all three conditions resulted in reduced microbial growth relative to the control. Specifically, decreases of 1.42, 1.20, and 1.44 log CFU/mL were observed in the presence of GCA, TCA, and TDCA, respectively.

In contrast, the bioreactor culture exhibited statistically significant differences in the growth of *L. fermentum* K73 among the three bile salts evaluated (*p* = 0.0006). Tukey’s *post hoc* analysis revealed that TCA supported the highest microbial proliferation, significantly exceeding the values observed with TDCA (*p* = 0.0042) and GCA (*p* = 0.0006). Additionally, TDCA induced significantly greater growth than GCA (*p* = 0.0043). This behavior may be attributed to enhanced bile salt metabolism under the acidic conditions generated during fermentation and aligns with observations reported by Gil-Rodríguez and Beresford (2021), who emphasized the role of pH reduction in promoting bile salt precipitation and microbial utilization.

For the C1 formulation in its dissolved state, statistically significant differences were also identified among the bile salts (*p* < 0.001). No microbial growth was detected in the presence of GCA, while viable counts were maintained with TCA and TDCA. TCA exhibited the highest bacterial load, significantly greater than TDCA.

Similarly, the C2 formulation showed significant differences (*p* < 0.001), with both TCA and TDCA supporting higher growth than GCA (*p* < 0.001). No significant difference was observed between TCA and TDCA (*p* = 0.101). In the case of C3, all pairwise comparisons revealed significant differences (*p* < 0.001). TCA induced the highest growth, followed by TDCA, which was significantly higher than GCA, with TCA also outperforming TDCA.

Together, these results consistently demonstrate that *L. fermentum* K73 clearly prefers tauroconjugated bile salts, particularly TCA, across all tested conditions and encapsulation systems.

It is important to note that encapsulation does not inherently impede BSH activity. In fact, previous studies have reported enhanced enzyme functionality in encapsulated probiotics. For instance, microencapsulation of *Lactobacillus reuteri* increased its BSH activity, likely due to bile salt diffusion through microcapsule pores (Martoni et al., 2008; Kumar et al., 2011). Notably, while *L. reuteri* has shown greater affinity for GCA, *L. fermentum* K73 in this study demonstrated superior interaction with TCA and TDCA, highlighting strain-specific enzymatic behaviors that must be accounted for during probiotic selection and formulation.

In the digested C1 formulation, a shift in behavior was observed. No statistically significant differences were detected among the bile salts; however, microbial growth in GCA increased relative to prior stages. This may relate to findings by Martoni et al. (2007), who noted enhanced enzymatic response to GCA in *L. plantarum* following *in vitro* digestion. Their hypothesis suggests that enzyme-substrate binding post-deconjugation reduces intestinal absorption, promoting excretion in the intestinal phase. Though this mechanism differs from that observed here, it underscores the role of digestion-induced physiological changes in modulating BSH activity.

Moreover, the improved growth in TCA relative to the control may be influenced by the encapsulation matrix. As reported by Zhou et al. (2024), double emulsions not only provide superior probiotic protection but also enhance the bioavailability of active compounds. Likewise, Melchior et al. (2022) emphasized the role of whey proteins in forming protective protein clots during the gastric phase, allowing controlled probiotic release during intestinal transit, which may have contributed to the performance of C1 in this study.

Overall, the three encapsulation strategies demonstrated the ability to preserve the strain’s bile salt deconjugation capacity and viability, validating their potential as delivery vehicles for functional foods. Notably, C1 outperformed the other formulations in preserving viability under bile salt exposure and digestive stress, reinforcing its suitability for probiotic applications targeting cholesterol management. These findings highlight the resilience and versatility of *L. fermentum* K73 as a promising candidate for nutraceutical development.

Future work will explore *in vivo* validation using recombinant *bsh*-expressing clones and microencapsulated formulations in animal models to deepen the understanding of its hypocholesterolemic mechanism. Quantitative assessment of *bsh* gene expression via qPCR will further elucidate the enzymatic dynamics involved in cholesterol modulation.

### 3.4 Cholesterol absorption

**Figure 2:**
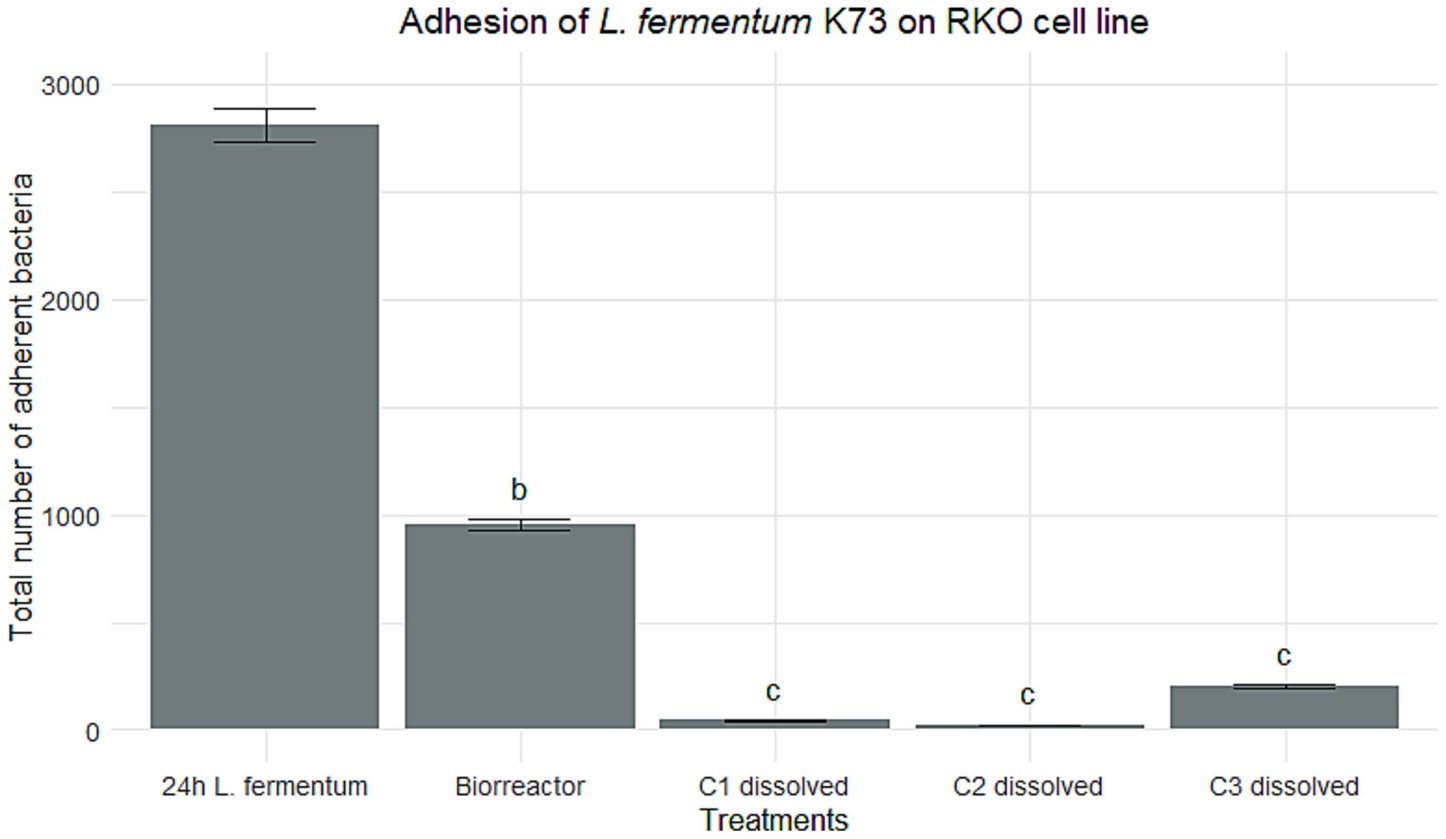
Cholesterol calibration curve. Calibration curve for the determination of cholesterol. Absorbance (ABS) is plotted as a function of cholesterol concentration (μg/mL). The dots represent the average of the measurements with their respective standard error bars. The equation of the linear regression line obtained was y = 0.005x + 0.3187, with a coefficient of determination *R*^2^ = 0.9503, indicating a high linear correlation between the variables.

To assess the cholesterol absorption capacity of *Limosilactobacillus fermentum* K73, a calibration curve was established using cholesterol concentrations ranging from 10 to 70 μg/mL (Figure 2). Based on prior findings indicating superior performance under simulated gastrointestinal digestion, only the C1 encapsulate was selected for further evaluation in the post-digestion stage, including the assessment of cholesterol removal and the presence of the *bsh* gene.

As shown in Table 3, *L. fermentum* K73 demonstrated cholesterol absorption capacities of 63.9% in 24-h MRS broth culture and 62.3% in the bioreactor, corresponding to 44.67 and 43.56 μg/mL of absorbed cholesterol, respectively. These values are slightly lower than those Aragón Rojas (2012) reported, who documented an absorption of 53.06 μg/mL (75.8%) by the same strain. Among the three encapsulation strategies tested, C1 exhibited the most favorable outcome, achieving an absorption of 23.74 μg/mL (33.91%) in its dissolved state, outperforming C2 and C3.

**Table 3:** Concentration and percentage of absorbed cholesterol.

Measurement points	Absorbed cholesterol /mL	Percentage of cholesterol absorbed %
24 h *L. fermentum* broth	44.74 ± 0.05	63.9
Bioreactor	43.64 ± 0.04	62.3
C1 dissolved	23.74 ± 0.04	33.91
C2 dissolved	23.44 ± 0.02	33.49
C3 dissolved	20.54 ± 0.03	29.34
C1 digest	45.84 ± 0.1	65.49

These results suggest that the encapsulation process, while critical for ensuring survival during transit, may impose a trade-off on probiotic functionality, possibly due to incomplete release of viable cells during *in vitro* decapsulation Zhou et al. (2024). Despite this, the C1 formulation demonstrated remarkable recovery post-digestion. Following the simulated intestinal phase, C1 exhibited the highest cholesterol uptake across all experimental conditions, reaching 45.84 μg/mL, equivalent to 65.49% absorption.

This improvement may be attributed to the complete release of viable probiotic cells facilitated by enzymatic activity and matrix disintegration in the intestinal phase. According to Melchior et al. (2022), digestion-mediated release mechanisms, particularly those involving protein clots formed by encapsulation matrices such as whey proteins, can enhance probiotic bioactivity. Furthermore, Padhmavathi et al. (2023) and Asgari et al. (2020) emphasize that the compatibility of wall materials with gastrointestinal conditions is crucial in safeguarding probiotic integrity while enabling functional performance.

Additionally, the cholesterol-lowering effect observed may be mechanistically linked to BSH-mediated bile salt deconjugation. Öner et al. (2014) reported that strains such as *Bifidobacterium longum* and *Lactobacillus plantarum* could precipitate cholesterol in bile salt-enriched media due to their deconjugation of taurocholic acid (TCA), leading to reduced cholesterol solubility and enhanced removal. The current study suggests a similar mechanism may be at play in *L. fermentum* K73, which showed greater affinity for TCA and TDCA. These findings reinforce the hypothesis that the enhanced cholesterol absorption observed post-digestion in C1 may be driven by effective deconjugation of tauroconjugated bile salts and subsequent cholesterol precipitation.

### 3.5 Molecular identification of the presence of the BSH enzyme

**Figure 3:**
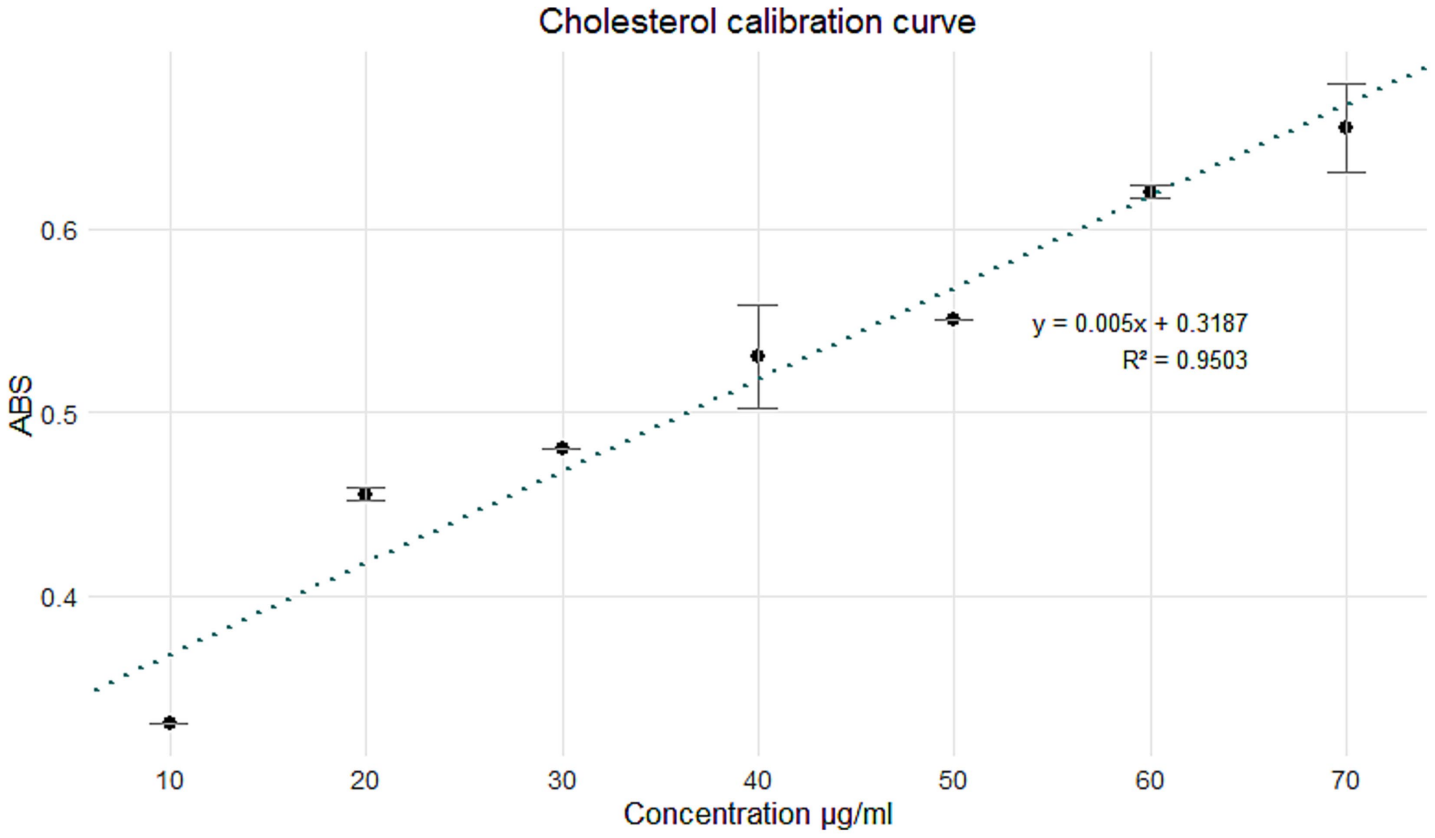
1.5% agarose gel electrophoresis of cDNA amplified in conventional PCR. The Figure shows the *bsh* gene amplifications for strains lane 1 ATCC *Limosilactobacillus fermentum* as a positive control, lane 2 24 h *L. fermentum* broth, lane 3 bioreactor, lane 4 C1 dissolved, lane 5 C1 digest, and lane 6 ATCC *Lactobacillus plantarum* as a negative control. *MP: molecular weight marker.

The presence of the *bsh* gene, with an expected amplicon size of approximately 638 bp, was successfully confirmed through conventional PCR in both control and experimental samples, corroborating the results obtained in the semi-qualitative bile salt deconjugation assay and the cholesterol absorption analysis. The amplification reactions yielded clean and distinct bands without evidence of nonspecific products or primer dimers, confirming the specificity of the primers and the integrity of the cDNA used (Figure 3).

Significantly, the gene was consistently amplified from *L. fermentum* K73 across different stages of processing, including growth in MRS broth (Figure 3, Lane 2), fermentation in a bioreactor (Lane 3), and encapsulation via spray drying in capsule formulation C1 (Lane 4). These findings suggest that the strain retains the *bsh* gene in its genome regardless of the applied biotechnological processes and over time, indicating genetic stability and functional conservation of this cholesterol-lowering mechanism.

In line with these observations, Jayashree et al. (2014) reported the presence of two *bsh*-related genes, *bsh1* and *bsh2*, in another *L. fermentum* strain, with larger amplicon sizes of 927 bp and 978 bp, respectively. Similarly, Liang et al. (2018) identified homologous *bsh* sequences across seven complete *L. fermentum* genomes through *in silico* analysis. Complementary to these findings, Rani et al. (2017) described a 975 bp *bsh* gene in *Lactobacillus gasseri* FR4, which includes a conserved catalytic cysteine residue (Cys1) crucial for enzymatic activity in the presence of bile salts. These studies support the diversity of *bsh* gene variants among *Lactobacillus* species and highlight their role in bile salt metabolism and microbial adaptation to the intestinal environment.

However, it is notable that in Figure 3, Lane 5—corresponding to C1 post-digestion—no amplification product was detected, indicating an absence of detectable *bsh* gene expression following simulated gastrointestinal digestion. This phenomenon may be attributed to the physiological stress encountered during digestion, such as drastic pH fluctuations, exposure to bile salts, and enzymatic activity, which may impair cellular function or reduce transcriptional activity as part of a survival strategy (Foster, 2005). Han et al. (2021) similarly emphasized that gastrointestinal transit conditions can compromise microbial cell integrity and DNA stability, potentially leading to reduced gene expression or damage to genetic material.

These results underline the necessity of incorporating gene expression quantification techniques, such as real-time quantitative PCR (qPCR), in future studies. Such approaches would enable not only confirmation of gene presence but also assessment of transcriptional dynamics under different physiological conditions, providing a more complete understanding of the role of *bsh* in probiotic function.

### 3.6 Adhesion to intestinal cells by *in vitro* assays

**Figure 4:**
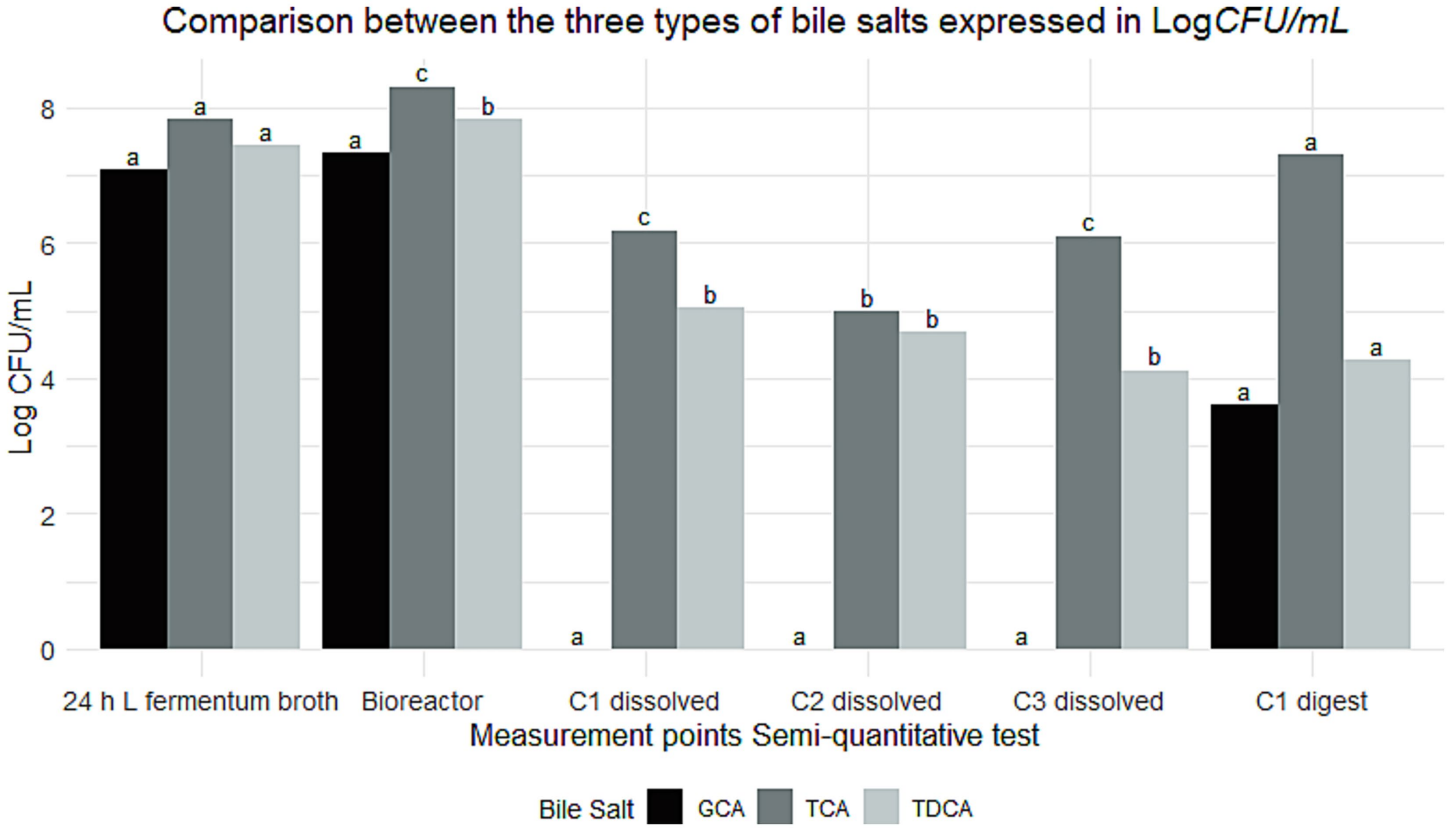
The following study will examine the adherence of *Limosilactobacillus fermentum* K73 to the HT-29 cell line under different treatments. The bars represent the total number of adherent bacteria per treatment. The error bars represent the standard deviation. Letters above the bars denote statistical significance groups obtained by a post-hoc test (specify which one if known, e.g., Tukey, *p* < 0.05). Treatments marked with different letters demonstrate significant disparities in bacterial adhesion.

The adhesion capacity of *Limosilactobacillus fermentum* K73 to intestinal epithelial cells was evaluated using two cell lines: HT-29 and RKO. The adhered bacteria were quantified according to the protocol described by Chauviere et al. (1992). The results obtained for the HT-29 cell line are shown in Figure 4.

The highest adhesion rate to HT-29 cells was recorded in the 24-h broth culture, with 2,488 ± 24.11 adhered to the bacteria. This value exceeds those reported for *Lactiplantibacillus plantarum* strains, which exhibited adhesion levels of approximately 800 (Muryany et al., 2018). It is also more consistent with results from Zawistowska-Rojek et al. (2022), who observed values ranging from 1800 to 2,200. These findings confirm the strong adhesion potential of *L. fermentum* K73 in its unencapsulated form.

Following encapsulation and subsequent processing, a significant reduction in adhesion was observed across treatments. ANOVA confirmed statistically significant differences between treatments (*p* < 0.001), and Tukey’s *post hoc* test indicated that adhesion in the 24 h broth was significantly higher than all other groups (*p* < 0.001). The bioreactor treatment retained moderate adhesion (195 ± 8.10), while C1, C2, and C3 dissolved capsules showed reduced values of 21 ± 0.72, 5 ± 0.25, and 33 ± 0.81, respectively. After *in vitro* digestion, the encapsulated formulations showed partial recovery of adhesive potential, with C1 digest: 41 ± 0.79, C2 digest: 61 ± 1.04, and C3 digest: 45 ± 0.90. These values were significantly higher than their respective dissolved forms (*p* < 0.01), highlighting the protective effect of encapsulation and the release of viable adhesive cells upon digestion.

This reduction in adhesion may be attributed to interactions between the probiotic and matrix proteins, particularly whey proteins. According to Zhu et al. (2021), these interactions may lead to flocculation, reducing surface protein exposure and impairing adhesion. Similarly, Hsein et al. (2015) observed that matrix components may modulate covalent interactions between bacterial adhesins and epithelial receptors. Additionally, peptides released during the digestion of whey proteins may exert antimicrobial effects, inhibiting pathogen adhesion while supporting probiotic colonization (Morrissey et al., 2010).

Burgain et al. (2013) demonstrated that protein structure and microcapsule architecture influence specific interactions between *Lactobacillus rhamnosus* GG and dairy proteins. They proposed that bacteria tend to localize at the fat–protein interface, particularly when displaced during network formation, further affecting adhesion efficiency. The observations in the current study reinforce the hypothesis that encapsulation matrix composition and digestion processes influence bacterial adhesion properties *in vitro*.

**Figure 5:**
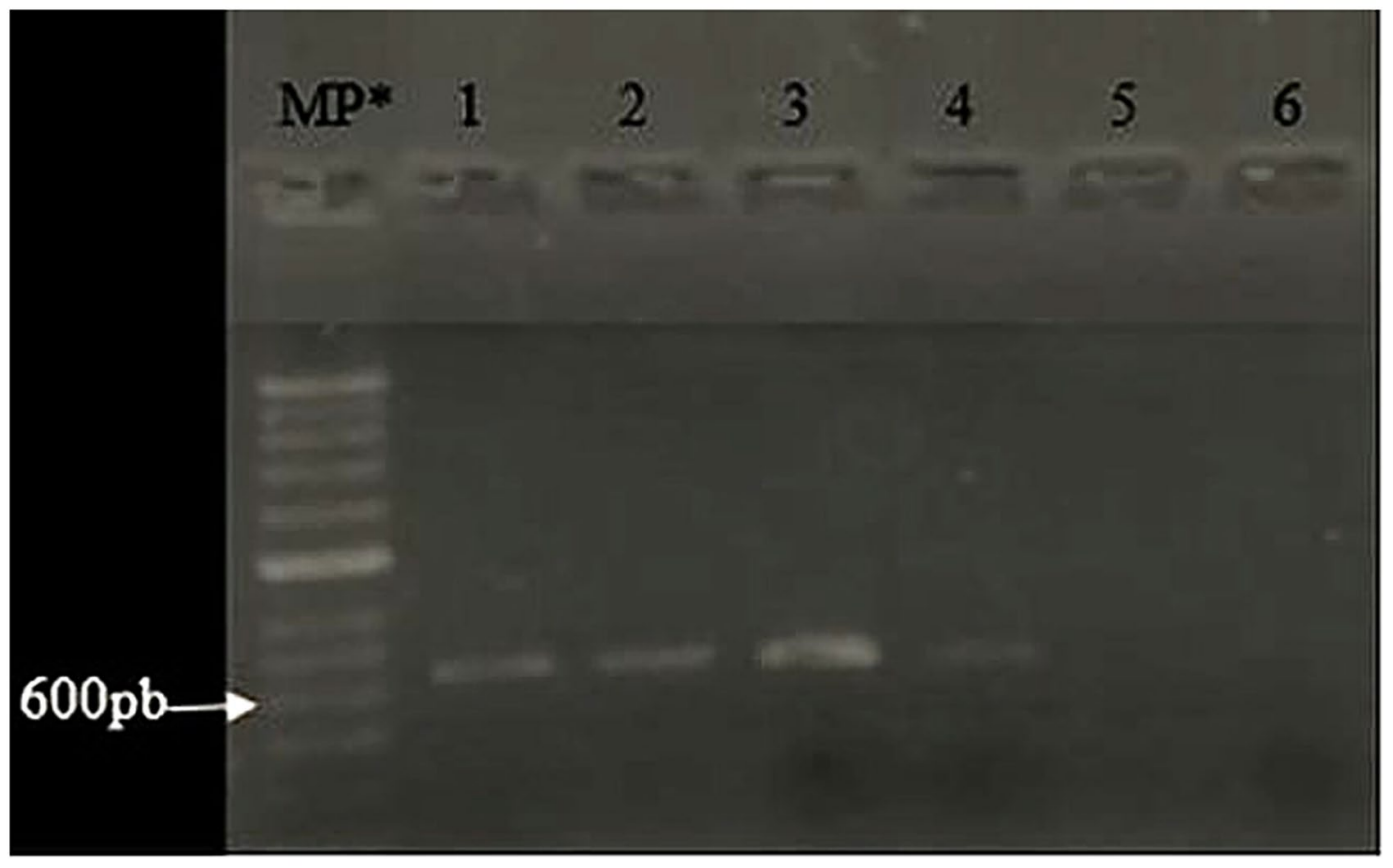
The following study investigates the total adhesion of *Limosilactobacillus fermentum* K73 to the RKO cell line under different treatments. The bars represent the sum of bacteria counts per treatment, and the error bars indicate the standard deviation. Letters above the bars denote statistical significance groups obtained by Tukey’s test (*p* < 0.05). Treatments marked with different letters demonstrate significant disparities in bacterial adhesion.

The RKO cell line, representing early-stage colorectal carcinoma, was also employed to broaden the evaluation. This line is recognized for its genetic plasticity and suitability in mechanistic studies involving gene expression and cellular response. As shown in Figure 5, *L. fermentum* K73 exhibited higher adhesion in RKO cells than HT-29 for equivalent treatments. The 24 h broth recorded 2,806 ± 28.71 adhered cells, followed by bioreactor (952 ± 11.44), and C1, C2, and C3 dissolved (44 ± 1.21, 22 ± 0.53, and 201 ± 4.80, respectively). ANOVA confirmed significant differences between treatments (*p* < 0.001), and Tukey’s *post hoc* analysis revealed that all pairwise comparisons between treatments were statistically significant (*p* < 0.05), except between C1 and C2 dissolved forms.

The greater adhesion observed in RKO cells may be due to differences in surface receptor expression and epithelial maturity compared to HT-29. However, adhesion assessments post-digestion were not feasible in RKO due to enzymatic degradation of the cell monolayer, which led to detachment from the 96-well plates. This limitation is likely related to RKO cells’ immature and fragile differentiation status.

Nonetheless, RKO cells are widely used in adhesion studies due to their altered expression of fibronectin and other adhesive ligands (Tarapatzi et al., 2022). Optimizing enzyme inactivation or protective layering strategies in future studies could preserve epithelial integrity, thereby allowing for comprehensive evaluation of post-digestion adhesion under simulated gastrointestinal conditions.

## 4 Conclusion

This study confirmed that *Limosilactobacillus fermentum* K73 retains its hypocholesterolemic activity throughout the various processing stages, including fermentation, encapsulation, and simulated gastrointestinal digestion. The strain consistently demonstrated the ability to deconjugate bile salts, likely through bile salt hydrolase (BSH) activity, and exhibited efficient cholesterol absorption. Among the three encapsulation strategies evaluated, microcapsule C1 showed superior performance, particularly in survival during *in vitro* digestion, tolerance to bile salts, and cholesterol removal, positioning it as the most effective delivery system for maintaining probiotic functionality. These advantages were further supported by the higher viable cell counts and cholesterol absorption observed in digested C1 samples compared to the other encapsulated formulations.

Additionally, *in vitro* assays demonstrated moderate adhesion of *L. fermentum* K73 to intestinal epithelial cells, suggesting that the encapsulation process does not hinder, and may even support, its capacity for epithelial interaction—a key factor for colonization and functional efficacy in the host. These findings position microcapsule 1 (C1) as a promising formulation for incorporation into functional food products aimed at cholesterol management.

However, the study also recognizes certain methodological limitations. The adhesion assays performed under *in vitro* conditions may not fully capture the strain’s interaction dynamics within the intestinal environment. Thus, future studies should consider more advanced or complementary adhesion models to better characterize these interactions. Moreover, while BSH activity was inferred based on phenotypic outcomes, the amplification of the *bsh* gene during post-digestion analysis was unsuccessful. To address this, we propose using quantitative real-time PCR (qPCR) in future work to confirm gene presence and quantify its expression. This would enable a deeper understanding of the enzymatic mechanisms involved and further validate *L. fermentum* K73 as a robust candidate for hypocholesterolemic probiotic applications.

## Data Availability

The original contributions presented in the study are included in the article/supplementary material, further inquiries can be directed to the corresponding author/s.

## References

[ref1] Agudelo-Chaparro J. Ciro-Velásquez H. J. Sepúlveda-Valencia J. U. Pérez-Monterroza E. J. (2022). Microencapsulation of *Lactobacillus rhamnosus* ATCC 7469 by spray drying using maltodextrin, whey protein concentrate and trehalose. Food Sci. Technol. Int. 28, 476–488. doi: 10.1177/10820132211020621, PMID: 34058894

[ref2] Aragón Rojas S. (2012) Evaluación bioquímica y molecular de la capacidad hipocolesterolémica de bacterias ácido lácticas con potencial probiótico. Universidad de La Sabana. Available at: http://hdl.handle.net/10818/8321 (Accessed November 2, 2012).

[ref3] Aragón-Rojas S. Hernández-Álvarez A. J. Mainville I. Arcand Y. Quintanilla-Carvajal M. X. (2020). Effect of the carrier material, drying technology and dissolution media on the viability of *Lactobacillus fermentum* K73 during simulated gastrointestinal transit. Food Funct. 11, 2339–2348. doi: 10.1039/c9fo01091b, PMID: 32118211

[ref4] Aragón-Rojas S. Quintanilla-Carvajal M. X. Hernández-Sánchez H. (2018). Multifunctional role of the whey culture medium in the spray-drying microencapsulation of lactic acid Bacteria. Food Technol. Biotechnol. 56, 381–397. doi: 10.17113/ftb.56.03.18.5285, PMID: 30510482 PMC6233008

[ref5] Aragón-Rojas S. Quintanilla-Carvajal M. X. Hernández-Sánchez H. Hernández-Álvarez A. J. Moreno F. L. (2019). Encapsulation of *Lactobacillus fermentum* K73 by Refractance window drying. Sci. Rep. 9, 5625–5615. doi: 10.1038/s41598-019-42016-0, PMID: 30948743 PMC6449500

[ref6] Archer A. C. Kurrey N. K. Halami P. M. (2018). *In vitro* adhesion and anti-inflammatory properties of native *Lactobacillus fermentum* and *Lactobacillus delbrueckii* spp. J. Appl. Microbiol. 125, 243–256. doi: 10.1111/jam.13757, PMID: 29537703

[ref7] Asgari S. Pourjavadi A. Licht T. R. Boisen A. Ajalloueian F. (2020). Polymeric carriers for enhanced delivery of probiotics. Adv. Drug Deliv. Rev. 161-162, 1–21. doi: 10.1016/j.addr.2020.07.014, PMID: 32702378

[ref9001] Bauer Estrada K. Cardoso Cardenas M. Carreño Cuellar M. Quintanilla-Carvajal M. X. (2025). Enhancing the Gastrointestinal Survival of Microencapsulated Limosilactobacillus fermentum K73: Optimization Through Double Emulsification Followed by Spray Drying, and in Vitro Digestion Assessment. Food Biophysics, 20, 1–15. doi: 10.1007/S11483-025-09975-6/FIGURES/3

[ref8] Bennacef C. Desobry-Banon S. Probst L. Desobry S. (2021). Advances on alginate use for spherification to encapsulate biomolecules. Food Hydrocoll. 118:6782. doi: 10.1016/j.foodhyd.2021.106782

[ref9] Bhagwat A. Bhushette P. Annapure U. S. (2020). Spray drying studies of probiotic *Enterococcus* strains encapsulated with whey protein and maltodextrin. Beni Suef Univ. J. Basic Appl. Sci. 9:61. doi: 10.1186/s43088-020-00061-z

[ref10] Burgain J. Gaiani C. Francius G. Revol-Junelles A. M. Cailliez-Grimal C. Lebeer S.. (2013). In vitro interactions between probiotic bacteria and milk proteins probed by atomic force microscopy. Colloids Surf. B: Biointerfaces 104, 153–162. doi: 10.1016/j.colsurfb.2012.11.032, PMID: 23298601

[ref11] Chauviere G. Coconnier M. H. Kernéis S. Fourniat J. Servin A. L. (1992). Adhesion of human *Lactobacillus acidophilus* strain LB to human enterocyte-like Caco-2 cells. J. Gen. Microbiol. 138, 1689–1696. doi: 10.1099/00221287-138-8-1689 1527509

[ref12] Cueto C. Aragón S. (2012). Evaluación del potencial probiótico de bacterias ácido lácticas para reducir el colesterol in vitro Evaluation of probiotic potential of lactic acid bacteria to reduce in vitro cholesterol. Sci. Agropec. 1, 45–50. doi: 10.17268/sci.agropecu.2012.01.06

[ref13] Da Fonseca A. M. dos Santos J. C. S. de Souza M. C. M. de Oliveira M. M. Colares R. P. de Lemos T. L. G.. (2020). The use of new hydrogel microcapsules in coconut juice as biocatalyst system for the reaction of quinine. Ind. Crop. Prod. 145:111890. doi: 10.1016/j.indcrop.2019.111890

[ref14] Dong Z. Lee B. H. (2018). Bile salt hydrolases: structure and function, substrate preference, and inhibitor development. Protein Sci. 27, 1742–1754. doi: 10.1002/pro.3484, PMID: 30098054 PMC6199152

[ref15] Dos Santos Nascimento D. Sampaio K. B. do Nascimento Y. M. de Souza T. A. de Souza F. S. Júnior J. V. C.. (2024). Evaluating the stability of a novel nutraceutical formulation combining probiotic *Limosilactobacillus fermentum* 296, quercetin, and resveratrol under different storage conditions. Probiotics Antimicrob. Proteins 16, 13–25. doi: 10.1007/s12602-022-10011-z, PMID: 36417111

[ref16] Eratte D. Dowling K. Barrow C. J. Adhikari B. P. (2017). *In-vitro* digestion of probiotic bacteria and omega-3 oil co-microencapsulated in whey protein isolate-gum Arabic complex coacervates. Food Chem. 227, 129–136. doi: 10.1016/j.foodchem.2017.01.080, PMID: 28274412

[ref17] Foster P. L. (2005). Stress responses and genetic variation in bacteria. Mutat. Res. 569, 3–11. doi: 10.1016/j.mrfmmm.2004.07.017, PMID: 15603749 PMC2729700

[ref9002] Gil-Rodríguez A. M. Beresford T. (2021). Bile salt hydrolase and lipase inhibitory activity in reconstituted skim milk fermented with lactic acid bacteria, Journal of Functional Foods, 77:104342. doi: 10.1016/j.jff.2020.104342

[ref18] Gullifa G. Risoluti R. Mazzoni C. Barone L. Papa E. Battistini A.. (2023). Microencapsulation by a spray drying approach to produce innovative probiotics-based products extending the shelf-life in non-refrigerated conditions. Molecules 28:860. doi: 10.3390/molecules28020860, PMID: 36677918 PMC9862012

[ref19] Han S. Lu Y. Xie J. Fei Y. Zheng G. Wang Z.. (2021). Probiotic gastrointestinal transit and colonization after Oral administration: a long journey. Front. Cell. Infect. Microbiol. 11:609722. doi: 10.3389/fcimb.2021.609722, PMID: 33791234 PMC8006270

[ref20] Hsein H. Garrait G. Beyssac E. Hoffart V. (2015). Whey protein mucoadhesive properties for oral drug delivery: mucin-whey protein interaction and mucoadhesive bond strength. Colloids Surf. B: Biointerfaces 136, 799–808. doi: 10.1016/j.colsurfb.2015.10.016, PMID: 26529388

[ref21] Huang C. H. Ho C. Y. Chen C. T. Hsu H. F. Lin Y. H. (2019). Probiotic BSH activity and anti-obesity potential of *Lactobacillus plantarum* strain TCI378 isolated from Korean kimchi. Prev. Nutr. Food Sci. 24, 434–441. doi: 10.3746/pnf.2019.24.4.434, PMID: 31915639 PMC6941724

[ref22] Jayashree S. Pooja S. Pushpanathan M. Rajendhran J. Gunasekaran P. (2014). Identification and characterization of bile salt hydrolase genes from the genome of *Lactobacillus fermentum* MTCC 8711. Appl. Biochem. Biotechnol. 174, 855–866. doi: 10.1007/s12010-014-1118-5, PMID: 25099376

[ref23] Jiang J. Ma C. Song X. Zeng J. Zhang L. Gong P. (2022). Spray drying co-encapsulation of lactic acid bacteria and lipids: a review. Trends Food Sci. Technol. 129, 134–143. doi: 10.1016/j.tifs.2022.09.010

[ref24] Kimoto H. Ohmomo S. Okamoto T. (2002). Cholesterol removal from media by Lactococci. J. Dairy Sci. 85, 3182–3188. doi: 10.3168/jds.S0022-0302(02)74406-8, PMID: 12512591

[ref25] Kumar R. Grover S. Batish V. K. (2011). Hypocholesterolaemic effect of dietary inclusion of two putative probiotic bile salt hydrolase-producing *Lactobacillus plantarum* strains in Sprague–Dawley rats. Br. J. Nutr. 105, 561–573. doi: 10.1017/S0007114510003740, PMID: 20923582

[ref26] Latif A. Shehzad A. Niazi S. Zahid A. Ashraf W. Iqbal M. W.. (2023). Probiotics: mechanism of action, health benefits and their application in food industries. Front. Microbiol. 14:674. doi: 10.3389/fmicb.2023.1216674, PMID: 37664108 PMC10470842

[ref27] Liang L. Yi Y. Lv Y. Qian J. Lei X. Zhang G. (2018). A comprehensive genome survey provides novel insights into bile salt hydrolase (BSH) in Lactobacillaceae. Molecules 23:1157. doi: 10.3390/molecules23051157, PMID: 29751655 PMC6100381

[ref28] Liu H. Cui S. W. Chen M. Li Y. Liang R. Xu F.. (2019). Protective approaches and mechanisms of microencapsulation to the survival of probiotic bacteria during processing, storage and gastrointestinal digestion: A review. Crit Rev Food Sci Nutr 59, 2863–2878. doi: 10.1080/10408398.2017.1377684, PMID: 28933562

[ref29] Liu H. Gong J. Chabot D. Miller S. S. Cui S. W. Ma J.. (2016). Incorporation of polysaccharides into sodium caseinate-low melting point fat microparticles improves probiotic bacterial survival during simulated gastrointestinal digestion and storage. Food Hydrocoll. 54, 328–337. doi: 10.1016/j.foodhyd.2015.10.016

[ref30] Lye H. S. Rusul G. Liong M. T. (2010). Removal of cholesterol by lactobacilli via incorporation and conversion to coprostanol. J. Dairy Sci. 93, 1383–1392. doi: 10.3168/jds.2009-2574, PMID: 20338415

[ref31] Markowiak-Kopeć P. Śliżewska K. (2020). The effect of probiotics on the production of short-chain fatty acids by human intestinal microbiome. Nutrients 12:1107. doi: 10.3390/nu12041107, PMID: 32316181 PMC7230973

[ref32] Martoni C. Bhathena J. Jones M. L. Urbanska A. M. Chen H. Prakash S. (2007). Investigation of microencapsulated BSH active Lactobacillus in the simulated human GI tract. J. Biomed. Biotechnol. 2007, 1–9. doi: 10.1155/2007/13684, PMID: 18273409 PMC2217584

[ref33] Martoni C. Bhathena J. Urbanska A. M. Prakash S. (2008). Microencapsulated bile salt hydrolase producing *Lactobacillus reuteri* for oral targeted delivery in the gastrointestinal tract. Appl. Microbiol. Biotechnol. 81, 225–233. doi: 10.1007/s00253-008-1642-8, PMID: 18719901

[ref34] Masco L. Crockaert C. van Hoorde K. Swings J. Huys G. (2007). In vitro assessment of the gastrointestinal transit tolerance of taxonomic reference strains from human origin and probiotic product isolates of Bifidobacterium. J. Dairy Sci. 90, 3572–3578. doi: 10.3168/jds.2006-548, PMID: 17638965

[ref35] Melchior S. Calligaris S. Marino M. D’Este F. Honsell G. Nicoli M. C.. (2022). Digestive protection of probiotic *Lacticaseibacillus rhamnosus* in ricotta cheese by monoglyceride structured emulsions. Int. J. Food Sci. Technol. 57, 3106–3115. doi: 10.1111/ijfs.15641

[ref36] Minekus M. Alminger M. Alvito P. Ballance S. Bohn T. Bourlieu C.. (2014). A standardised static in vitro digestion method suitable for food – an international consensus. Food Funct. 5, 1113–1124. doi: 10.1039/c3fo60702j, PMID: 24803111

[ref37] Mohammed N. K. Tan C. P. Manap Y. A. Muhialdin B. J. Hussin A. S. M. (2020). Spray drying for the encapsulation of oils—a review. Molecules 25:3873. doi: 10.3390/molecules25173873, PMID: 32858785 PMC7503953

[ref38] Monteagudo-Mera A. Rastall R. A. Gibson G. R. Charalampopoulos D. Chatzifragkou A. (2019). Adhesion mechanisms mediated by probiotics and prebiotics and their potential impact on human health. Appl. Microbiol. Biotechnol. 103, 6463–6472. doi: 10.1007/s00253-019-09978-7, PMID: 31267231 PMC6667406

[ref39] Moraffah F. Samadi N. Abdollahi M. Ostad S. N. Dolatabadi R. Pirouzzadeh M.. (2025). Advancing burn wound healing with an innovative in situ gelling probiotic microparticle formulation employing quality by design (QbD) principles. J. Tissue Viability 34:100860. doi: 10.1016/J.JTV.2025.100860, PMID: 39874740

[ref40] Morrissey P. E. W. Folan M. A. Baird A. W. Irwin J. A. (2010). Effects of a partially digested whey protein concentrate on *Salmonella enterica* serotype Typhimurium adhesion to Caco-2 cells: prevention of Salmonella adhesion to Caco-2 cells using whey. Food Control 21, 1113–1120. doi: 10.1016/j.foodcont.2010.01.005

[ref41] Muryany I. Lian H. H. Salwany I. S. Ghazali R. (2018). Adhesion ability and cytotoxic evaluation of *Lactobacillus* strains isolated from Malaysian fermented fish (Pekasam) on Ht-29 and Ccd-18Co intestinal cells (Keupayaan Perlekatan dan Penilaian Sitotoksik strain *Lactobacillus* Dipencilkan daripada Ikan Pekasam Malaysia ke atas Sel Usus Ht-29 dan Ccd-18Co). Sains Malaysiana 47, 2391–2399. doi: 10.17576/jsm-2018-4710-15

[ref42] Öner Ö. Aslim B. Aydaş S. B. (2014). Mechanisms of cholesterol-lowering effects of lactobacilli and Bifidobacteria strains as potential probiotics with their bsh gene analysis. J. Mol. Microbiol. Biotechnol. 24, 12–18. doi: 10.1159/000354316, PMID: 24158048

[ref43] Ouarabi L. Hamma-Faradji S. Barache N. Zidi G. Belguesmia Y. Drider D. (2025). Characterization of *Lactiplantibacillus plantarum* strains isolated from Algerian fruits for probiotic and biotechnological applications. Antonie Van Leeuwenhoek 118, 1–18. doi: 10.1007/s10482-025-02074-z, PMID: 40153083

[ref44] Padhmavathi V. Shruthy R. Preetha R. (2023). Chitosan coated skim milk-alginate microspheres for better survival of probiotics during gastrointestinal transit. J. Food Sci. Technol. 60, 889–895. doi: 10.1007/s13197-021-05179-1, PMID: 36908352 PMC9998793

[ref45] Puttarat N. Kasorn A. Vitheejongjaroen P. Chantarangkul C. Tangwattanachuleeporn M. Taweechotipatr M. (2023). Beneficial effects of indigenous probiotics in high-cholesterol diet-induced Hypercholesterolemic rats. Nutrients 15:2710. doi: 10.3390/nu15122710, PMID: 37375614 PMC10301077

[ref46] Raise A. Dupont S. Iaconelli C. Caliri C. Charriau A. Gervais P.. (2020). Comparison of two encapsulation processes to protect the commensal gut probiotic bacterium *Faecalibacterium prausnitzii* from the digestive tract. J. Drug Deliv. Sci. Technol. 56:101608. doi: 10.1016/j.jddst.2020.101608

[ref47] Rajam R. Subramanian P. (2022). Encapsulation of probiotics: past, present and future. Beni Suef Univ. J. Basic Appl. Sci. 11, 1–18. doi: 10.1186/s43088-022-00228-w, PMID: 40810051

[ref48] Ranadheera C. S. Evans C. A. Adams M. C. Baines S. K. (2012). In vitro analysis of gastrointestinal tolerance and intestinal cell adhesion of probiotics in goat’s milk ice cream and yogurt. Food Res. Int. 49, 619–625. doi: 10.1016/j.foodres.2012.09.007

[ref49] Rani R. P. Anandharaj M. Ravindran A. D. (2017). Characterization of bile salt hydrolase from *Lactobacillus gasseri* FR4 and demonstration of its substrate specificity and inhibitory mechanism using molecular docking analysis. Front. Microbiol. 8:1004. doi: 10.3389/fmicb.2017.01004/full 28620369 PMC5449720

[ref50] Ricaurte L. Correa R. E. P. de Jesus Perea-Flores M. Quintanilla-Carvajal M. X. (2017). Influence of milk whey on high-oleic palm oil nanoemulsions: powder production, physical and release properties. Food Biophys. 12, 439–450. doi: 10.1007/s11483-017-9500-9

[ref51] Rojas-Muñoz Y. V. Santagapita P. R. Quintanilla-Carvajal M. X. (2023). Probiotic encapsulation: bead design improves bacterial performance during in vitro digestion. Polymers 15:296. doi: 10.3390/polym15214296, PMID: 37959976 PMC10649307

[ref52] Rousseaux A. Brosseau C. Bodinier M. (2023). Immunomodulation of B lymphocytes by prebiotics, probiotics and Synbiotics: application in pathologies. Nutrients 15:269. doi: 10.3390/nu15020269, PMID: 36678140 PMC9863037

[ref53] Su X. Xu Y. Xu Z. Hurley K. Feng Y. Yin Y. (2023). Encapsulation of hop (*Humulus lupulus* L.) essential oil for controlled release in the non-alcoholic beverage application. Food Hydrocoll. 134:108039. doi: 10.1016/j.foodhyd.2022.108039

[ref54] Sudheer A. Dastidar D. G. Ghosh G. Taj Z. Nidhin I. K. Chattopadhyay I. (2025). Comprehensive genomics, probiotic, and antibiofilm potential analysis of *Streptococcus thermophilus* strains isolated from homemade and commercial dahi. Sci. Rep. 15, 7089–7029. doi: 10.1038/s41598-025-90999-w, PMID: 40016393 PMC11868508

[ref55] Tapía M. S. Alzamora S. M. Chirife J. (2020). “Effects of water activity (a w) on microbial stability as a hurdle in food preservation” in Water Activity in Foods: Fundamentals and Applications. eds. Barbosa-Cánovas G. V. Fontana A. J. Schmidt S. J. Labuza T. P. (Hoboken, NJ: Wiley), 323–355.

[ref56] Tarapatzi G. Filidou E. Kandilogiannakis L. Spathakis M. Gaitanidou M. Arvanitidis K.. (2022). The probiotic strains *Bifidοbacterium lactis*, *Lactobacillus acidophilus*, *Lactiplantibacillus plantarum* and *Saccharomyces boulardii* regulate wound healing and chemokine responses in human intestinal subepithelial myofibroblasts. Pharmaceuticals 15:1293. doi: 10.3390/ph15101293/s1 36297405 PMC9611312

[ref57] Vargas V. Saldarriaga S. Sánchez F. S. Cuellar L. N. Paladines G. M. (2024). Effects of the spray-drying process using maltodextrin on bioactive compounds and antioxidant activity of the pulp of the tropical fruit açai (*Euterpe oleracea* Mart.). Heliyon 10:e33544. doi: 10.1016/j.heliyon.2024.e33544, PMID: 39040403 PMC11260920

[ref58] Vega-Carranza A. S. Cervantes-Chávez J. A. Luna-Bárcenas G. Luna-González A. Diarte-Plata G. Nava-Mendoza R.. (2021). Alginate microcapsules as delivery and protective systems of *Bacillus licheniformis* in a simulated shrimp’s digestive tract. Aquaculture 540:736675. doi: 10.1016/j.aquaculture.2021.736675

[ref59] Vivek K. Mishra S. Pradhan R. C. Nagarajan M. Kumar P. K. Singh S. S.. (2023). A comprehensive review on microencapsulation of probiotics: technology, carriers and current trends. Appl. Food Res. 3:100248. doi: 10.1016/j.afres.2022.100248

[ref60] Yin M. Chen M. Yuan Y. Liu F. Zhong F. (2024). Encapsulation of *Lactobacillus rhamnosus* GG in whey protein isolate-shortening oil and gum Arabic by complex coacervation: enhanced the viability of probiotics during spray drying and storage. Food Hydrocoll. 146:109252. doi: 10.1016/j.foodhyd.2023.109252

[ref61] Zavaleta E. B. Coavichi L. I. L. Rodríguez L. C. V. Andrade E. F. García H. S. Rascón Díaz M. P. (2022). Co-microencapsulation of *Lactobacillus rhamnosus* and krill oil by spray-drying. Food Biosci. 50:102133. doi: 10.1016/j.fbio.2022.102133

[ref62] Zawistowska-Rojek A. Kośmider A. Stępień K. Tyski S. (2022). Adhesion and aggregation properties of Lactobacillaceae strains as protection ways against enteropathogenic bacteria. Arch. Microbiol. 204:285. doi: 10.1007/s00203-022-02889-8, PMID: 35478049 PMC9046290

[ref63] Zhou P. Li X. Jiang Z. Zhou J. Shen L. (2024). Facile construction of pectin-based hesperidin microcapsules: Solubilization, stability, loading process, and release mechanism. Food Chem. 451:139505. doi: 10.1016/j.foodchem.2024.139505, PMID: 38703732

[ref64] Zhou Y. Zhu L. Li Y. Guo F. Chen L. Wang G.. (2024). Encapsulation of probiotics (*Lactobacillus plantarum*) in soyasaponin–soybean protein isolate water-in-oil-in-water (W/O/W) emulsion for improved probiotic survival in the gastrointestinal tract. LWT 199:116100. doi: 10.1016/j.lwt.2024.116100

[ref65] Zhu X. Wang Q. Leng Y. Chen F. Wu F. Mu G.. (2021). Lecithin alleviates protein flocculation and enhances fat digestion in a model of infant formula emulsion. Food Chem. 346:128918. doi: 10.1016/j.foodchem.2020.128918, PMID: 33385913

